# Effects of increasing sensitizing doses of ovalbumin on airway hyperresponsiveness in asthmatic mice

**DOI:** 10.1002/iid3.1225

**Published:** 2024-03-27

**Authors:** Yan‐Jiao Chen, Yu Yuan, Lu Peng, Xin‐Yi Dong, Yu‐Dong Xu, Yu Wang, Yong‐Qing Yang

**Affiliations:** ^1^ Laboratory of Molecular Biology, Shanghai Research Institute of Acupuncture and Meridian Yueyang Hospital of Integrated Traditional Chinese and Western Medicine Shanghai P.R. China; ^2^ Deparment of Acupuncture and Moxibustion Guanghua Hospital Affiliated to Shanghai University of Traditional Chinese Medicine Shanghai P.R. China; ^3^ Shanghai University of Traditional Chinese Medicine Shanghai P.R. China

**Keywords:** airway resistance, allergen, asthma, inflammation

## Abstract

**Background:**

The dosage of ovalbumin (OVA) during the sensitization stage is considered a crucial factor in the development of airway hyperresponsiveness (AHR). However, the inconsistent dosages of sensitizing OVA used in current studies and the lack of research on their impact on AHR are notable limitations.

**Methods:**

We examined the impact of increasing sensitizing doses of OVA in a murine asthma model, which entailed initial sensitization with OVA followed by repeated exposure to OVA aerosols. BALB/c mice were primed with doses of OVA (0, 10, 20, 50, and 100 μg) plus 1 mg Alum on Days 0 and 7, and were challenged with OVA aerosols (10 mg/mL for 30 min) between Days 14 and 17. Antigen‐induced AHR to methacholine (MCh), as well as histological changes, eosinophilic infiltration, and epithelial injury were assessed.

**Results:**

The result indicated that there are striking OVA dose‐related differences in antigen‐induced AHR to MCh. The most intense antigen‐induced AHR to MCh was observed with sensitization at 50 μg, while weaker responses were seen at 10, 20, and 100 μg. Meanwhile, there was a significant increase in eosinophil count with sensitization at 50 μg. The changes of AHR were correlated with total cells count, lymphocytes count, eosinophils count, and basophils count in bronchoalveolar lavage fluid; however, it did not correlate with histological changes such as cellular infiltration into bronchovascular bundles and goblet cell hyperplasia of the bronchial epithelium.

**Conclusion:**

Overall, this study demonstrated that sensitization with 50 μg of OVA resulted in the most significant AHR compared to other dosages. These findings may offer valuable insights for future research on mouse asthma modeling protocols.

## 1 INTRODUCTION

Asthma is a complex disease characterized by airway hyperresponsiveness (AHR), chronic airway inflammation, and mucus overproduction.^1^ It currently affects more than 300 million people worldwide,^2^ with prevalence rates of almost 10% in high‐income countries and 2%–3% in low‐income countries among the adult population, representing a significant socioeconomic burden.^3^ As with most human diseases, animal models are essential tools for investigating the underlying mechanisms responsible for asthma. An appropriate animal model of asthma will greatly help researchers to explore novel mechanisms, elucidate complex interactions, and predict treatment responses with greater accuracy.

BALB/c mice are currently the most commonly used model to investigate asthma mechanisms. This strain of mouse can provide exuberant and appropriate AHR and Th2 responses that are notable in asthma.^4^ However, mice do not spontaneously develop asthma,^5^ and experimental strategies must be employed to induce the asthmatic‐like reaction to investigate the physiopathology of the disease. Mouse models for allergic asthma have been developed using various environmental risk factors, such as chicken egg ovalbumin (OVA), house dust mite, cockroach, and birch pollen. OVA is the most commonly utilized allergen for the induction of asthma in mouse models.^6^ However, despite being described as the “classical” murine allergic asthma model, there is no standardized OVA model due to variations in sensitization and challenge methods, OVA doses, use of alum, number of challenges, and protocol duration across studies.^7^, ^8^, ^9^, ^10^

The previous study has demonstrated that intraperitoneal injection of OVA and alum once a week for 2 weeks is the most straightforward and effective approach to induce an asthmatic reaction in mice.^11^ However, the inconsistent use of sensitizing OVA doses in current studies^12^, ^13^, ^14^ and the lack of research on their effect on AHR necessitate further investigation. Therefore, we conducted a study using increasing doses (0, 10, 20, 50, and 100 μg) of sensitizing OVA in a BALB/c murine model of asthma to evaluate their impact on AHR to methacholine (MCh), as well as histological changes, eosinophilic infiltration, and epithelial damage.

## 2 MATERIALS AND METHODS

### 2.1 Animals

Female BALB/c mice (6 weeks old, weighing 18 ± 2 g) were purchased from Beijing Vital River Laboratory Animal Technology Co. Ltd., and six mice per cage are housed under standard laboratory conditions with a temperature of 23 ± 1°C, humidity at 55 ± 5%, and free access to food and water under a 12 h light–dark cycle. All possible efforts were made to improve animal welfare and minimize the use of animals in the study.

### 2.2 Groups and asthma model

The main supervisor of the study, who allocated and conducted the experiment, was aware of the different experimental groups. Consequently, none of the individuals involved in data collection and analysis were informed about group allocation.

The 30 mice were allocated randomly into five distinct groups (six mice per group). We opted for a limited sample size based on previous research findings, which indicated that a sample size of six would be sufficient to detect significant differences between groups in the primary outcome measure AHR.^15^ The groups include a saline control (NS) group and four OVA groups with varying sensitizing doses of OVA. All mice were acclimatized for 1 week before experimentation. The control group mice were sensitized intraperitoneally and atomized with normal saline, while the other four OVA groups of mice were primed intraperitoneally with varying doses (10, 20, 50, or 100 μg) of OVA (Grade V; Sigma‐Aldrich) and 1 mg Imject Alum (Thermo) in 0.2 mL normal saline. Injections were given on Days 0 and 7. The sensitized mice were subjected to repeated exposure to an aerosol of OVA (10 mg/mL) delivered by a Compressor Nebulizer NB‐212C (SCIAN) driven by compressed air at a rate of 0.39 mL/min. Challenge was conducted for 30 min once daily for four consecutive days (Days 14–17). After 24 h of the final allergen exposure, mice were anesthetized, intubated, and subsequently subjected to measurement of AHR. Cages were assigned a numerical designation based on their position on the rack, and a cage was randomly selected from each group. The testing order of each animal within each group was randomized. The modeling and measurement methods employed in this study are based on the established approaches published in earlier research.^15^

### 2.3 Measurement of AHR

Changes in pulmonary functions were assessed in mice using the Buxco FinePointe resistance and compliance system (RC, DSI). Intraperitoneal injection of 1% pentobarbital sodium (Sigma‐Aldrich) at a dose of 100 mg/kg was administered as an anesthetic solution. After administering anesthesia, the mouse was secured onto the operating table. A precise incision was made in its trachea and intubation was carefully performed. Following the procedure, the mouse was transferred to a sealed detection chamber equipped with a thermostatic pad to maintain its body temperature.

Subsequently, mice were subjected to incremental doses of MCh (Sigma‐Aldrich) (0, 3.125, 6.25, 12.5, and 25.0 mg/mL) for challenge purposes while invasive resistance index (RI) was monitored at every interval of 2 s throughout the experiment to analyze AHR. The entire observation period lasts for 7 min for each concentration of MCh. After the experiment, euthanasia was performed on the mice by cervical dislocation, followed by subsequent sampling. All procedures were conducted in accordance with prescribed guidelines. The data was included in the study if the mice underwent successful measurement of AHR. The data was excluded if the mice died during the measurement.

### 2.4 Cell counting and analysis in bronchoalveolar lavage fluid (BALF)

The upper part of the main bronchus of the right lung was clamped using hemostatic forceps, and the alveolar space of the left lung was rinsed three times with 0.3 mL of cold phosphate‐buffered saline (PBS) (Hyclone) to collect the BALF. Each BALF sample was centrifuged at 1500 rpm for 5 min at 4°C, and the resulting cell pellet was resuspended in 160 μL of PBS. The total cell count, as well as counts for lymphocytes, macrophages, eosinophils, and basophils, were determined using a BC‐5300Vet counter (Mindray). The final analysis will include instrumentally tested and generated readable data samples only.

### 2.5 Histological evaluation of lung tissues

Lung tissue sections were prepared from each group of mice and subjected to hematoxylin and eosin (H&E; Beyotime Biotechnology) staining for eosinophil infiltration identification, as well as periodic acid‐Schiff (PAS; Beyotime Biotechnology) staining for mucus‐secreting goblet cell detection. The right lung of each mouse was removed and used for the preparation of pathological sections. The tissues were dissected, and fixed in 4% paraformaldehyde fix solution (MeilunBio) for 24 h. Following gradual dehydration and paraffin embedding, the tissues were sectioned into 4–5 μm thick slices. The paraffin‐embedded sections were carefully positioned on the staining rack. Before further processing, the samples required a deparaffinization and hydration procedure. This involved three rounds of xylene clearance, with each round lasting for 2 min. Followed by draining the xylene and hydrating the tissue section through decreasing concentrations of alcohol baths (100%, 95%, 70%) and water. Afterward, stained with H&E and PAS, and examined under a light microscope to evaluate histopathological alterations. The degree of lung inflammation was assessed by a treatment‐blinded observer using a subjective scale ranging from 0 to 4.^16^ The total lung inflammation was determined by calculating the average inflammation score across three lung sections per mouse, based on the inflammation scale (Table 1). The number of PAS‐stained mucin‐secreting cells in the airways was quantified by counting three lung bronchioles per mouse.^16^ The Goblet cell hyperplasia changes were quantified according to a modified 5‐point scoring system (grades 0–4) based on the percentage of goblet cells in the epithelium (Table 1).

**Table 1:** H&E and PAS staining score rules.

Score	H&E	Score	PAS
0	No inflammatory cells	0	No goblet cells
1	Occasionally inflammatory cells	1	<25% goblet cells
2	1–3 layer of inflammatory cells around bronchi or vessels	2	25%–50% goblet cells
3	4–5 layer of inflammatory cells around bronchi or vessels	3	51%–75% goblet cells
4	>5 layer of inflammatory cells around bronchi or vessels	4	>75% goblet cells

Abbreviations: H&E, hematoxylin and eosin; PAS, periodic acid‐Schiff.

### 2.6 Statistics analysis

The results were presented as the mean ± standard error of the mean (SEM). Statistical analyses were conducted using ANOVA with Tukey's post hoc correction using GraphPad Prism 8 Software. All group differences were considered statistically significant when *p* < .05.

## 3 RESULTS

### 3.1 OVA improved AHR in asthmatic mice model

**Figure 1:**
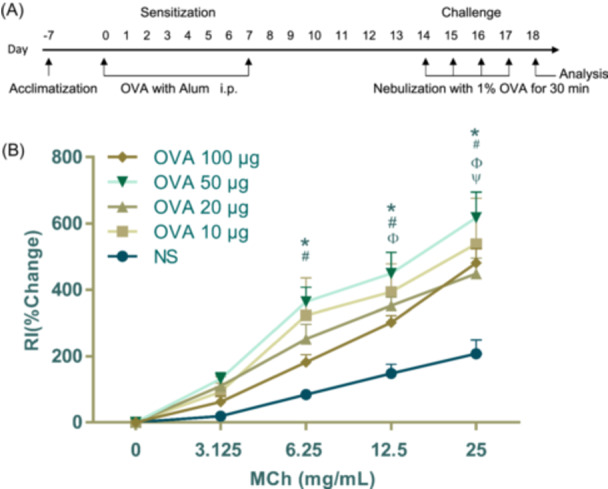
Development of sensitizing ovalbumin (OVA) doses induced airway hyperresponsiveness to methacholine (MCh). (A) Schedule of allergen sensitization and challenge in mice. (B) Airways resistance in each group. The values are presented as mean ± SEM (*n* = 6/group expect *n* = 5 in the OVA 10 μg group). *p < .05, OVA 50 μg versus NS challenged mice; ^#^ *p* < .05, OVA 10 μg versus NS challenged mice; ^Φ^ *p* < .05, OVA 20 μg versus NS challenged mice; ^ψ^ *p* < .05, OVA 100 μg versus NS challenged mice. *p* < .05 was considered as statistically significant.

Mice were sensitized and challenged with OVA or saline control as described in Section 2 (Figure 1A). After exposure to MCh at a concentration of 3.125 mg/mL, the RI exhibited an upward trend in the OVA groups with doses of 10, 20, 50, and 100 μg compared to the NS group, however, no statistically significant differences were observed among groups. Upon exposure to MCh at a concentration of 6.25 mg/mL, both the OVA 50 μg and OVA 10 μg groups exhibited a significant increase in RI compared to the NS group. Additionally, while there was no statistically significant difference between these two groups, the mean RI in the OVA 50 μg group was higher than that of the OVA 10 μg group. No significant difference in efficacy was observed between the OVA 20 μg and OVA 100 μg groups (Figure 1B). At the concentration of 12.5 mg/mL, the groups treated with OVA at doses of 50, 10, and 20 μg exhibited a significant ability to induce high respiratory resistance compared to the NS group. However, no statistically significant increase in RI was observed in the OVA 10 μg group. Moreover, all OVA challenge groups elicited a significant rise in airway resistance compared to the NS group at a concentration of 25 mg/mL (Figure 1B). The highest RI values were observed across all concentrations of MCh with sensitization at a dose of 50 μg. In the process of detection, we observed that at lower doses of MCh (e.g., 3.125 mg/mL), there was a high degree of uniformity among mice within each group. However, as the concentration of MCh increased, variations in data between groups became more pronounced.

### 3.2 OVA induced influx of immune cells in asthmatic mice model

**Figure 2:**
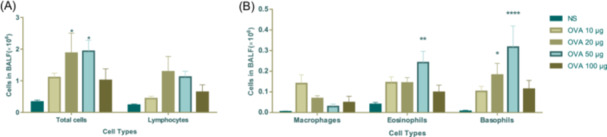
Ovalbumin (OVA)‐induced immune cells accumulation in bronchoalveolar lavage fluid (BALF). (A) Total cells count and lymphocytes in BALF. (B) Macrophages, eosinophils, and basophils in BALF. The values are presented as mean ± SEM (*n* = 5/6). *p < .05; **p < .01; ****p < .0001 versus the NS group. *p* < .05 was considered as statistically significant.

After the induction of allergic asthma, immune cells were collected from the BALF. The OVA 10, 20, 50, and 100 μg‐induced asthma groups exhibited an increase in total cell count by 331.7%, 563.4%, 581.4%, and 305.3%, respectively, compared to NS challenged mice (Figure 2A). Notably, the changes observed in OVA 20 and OVA 50 μg groups were statistically significant. The average number of lymphocyte cells in the OVA groups exhibited increases of 190.9%, 558.2%, 485.7%, and 278.6% compared to NS challenged mice, respectively; however, these increases were not statistically significant. Similarly, there was no statistical significance observed for changes in the average number of macrophage cells between the OVA groups and NS challenged mice (Figure 2A,B). Compared to the NS group, there was a significant increase on average eosinophil cell numbers by 364.9%, 361.1%, 611.1%, and 248.2% in the OVA‐induced asthma groups of 10, 20, 50, and 100 μg, respectively (Figure 2B). Only in the OVA‐induced asthma group of 50 μg were eosinophil cell numbers significantly higher than those observed in the NS group. The average basophils cell number was found to be significantly higher in the OVA‐induced asthma groups of both 20 and 50 μg compared to that in the NS group (Figure 2B).

### 3.3 OVA induced inflammation and mucin secretion in the lungs of the asthmatic mice model

**Figure 3:**
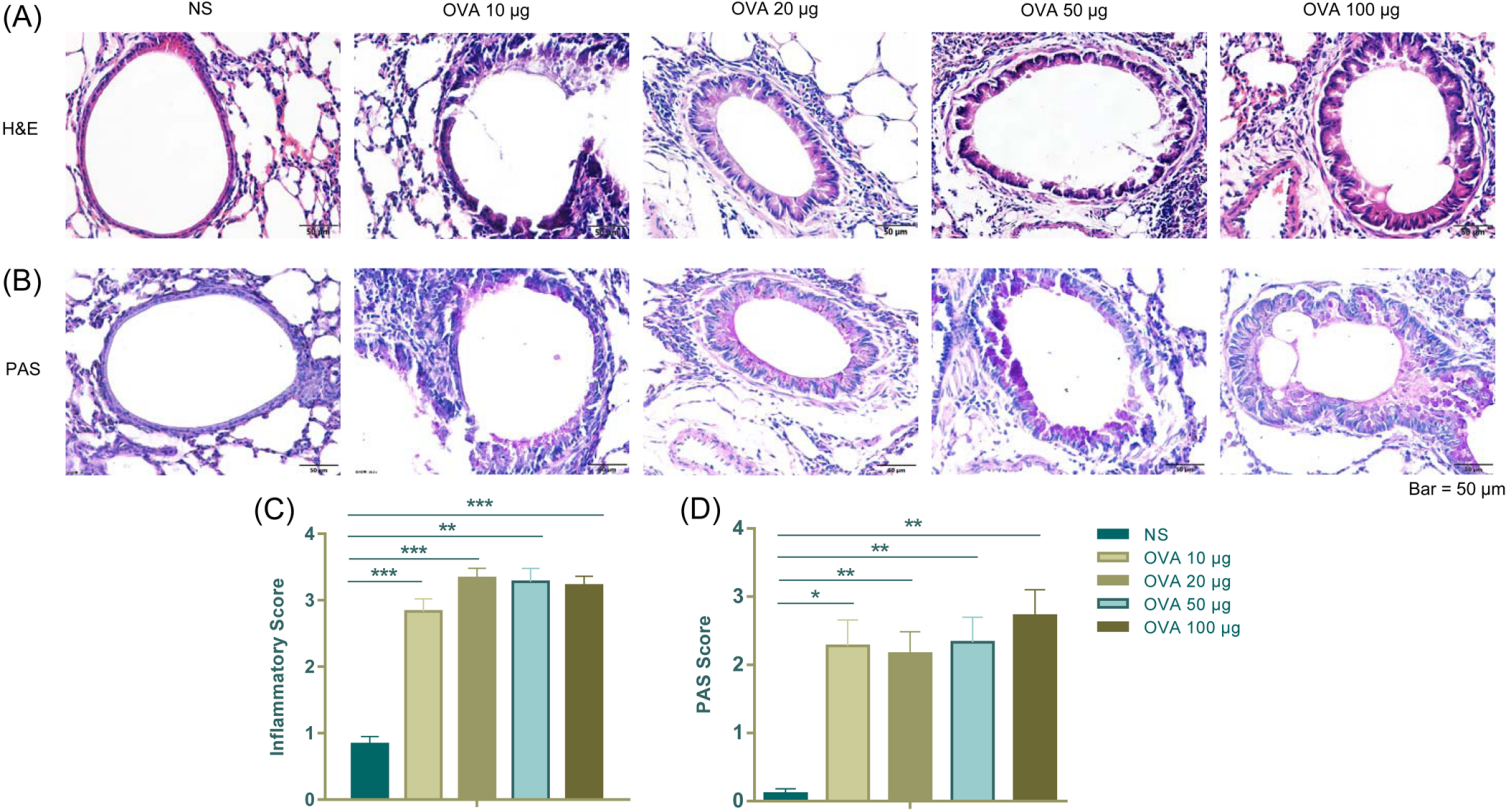
Ovalbumin (OVA)‐induced airway inflammation and mucin secretion in mice. (A) Hematoxylin and eosin (H&E)‐stained sections of lung tissues from the NS and OVA groups, small navy‐blue dots around the bronchioles indicate eosinophils. (B) Periodic acid‐Schiff (PAS)‐stained sections of lung tissues from the NS and OVA groups, mucin is stained as a purple color in PAS staining. (C) The inflammatory score of lung tissues from the NS and OVA groups. (D) The PAS score of lung tissues from the NS and OVA groups. The values are presented as mean ± SEM (*n* = 6). *p < .05; **p < .01; ***p < .001 versus the NS group. *p* < .05 was considered as statistically significant.

The degree of inflammation and mucin secretion was assessed through histological examination of lung tissues. The influx of eosinophils in the lung tissues was confirmed visually by H&E staining. H&E staining showed that no pathological changes were observed in the lungs of the NS group, while irregular arrangement of bronchial epithelial cells and a considerable number of inflammatory cells were observed in the OVA groups (Figure 3A).

Mucins were visualized through PAS staining. Violet‐colored mucins were infrequent in the NS group, while a darker and thicker purple coloring was observed surrounding the bronchiole in the OVA groups compared to the NS group (Figure 3B). The degree of lung inflammation and mucin production was analyzed using subjective scales as mentioned in Section 2.

There is a significant disparity observed between all OVA groups and the NS group in both H&E and PAS staining; however, no notable differences were found among the OVA 10, 20, 50, and 100 μg‐induced asthma groups (Figure 3C,D).

## 4 DISCUSSION

The sensitizing doses utilized in current murine studies typically range from 10 to 100 μg.^7^, ^17^, ^18^, ^19^ However, the effects of various doses within this range have not been thoroughly investigated. In 1999, Sakai et al.^20^ demonstrated the impact of sensitive OVA doses (0, 10, 100, and 1000 μg) on AHR; their study suggested that sensitization with a dose of 10 μg may be more effective than with a dose of 100 μg but almost completely inhibited at a dose of 1000 μg.

**Figure 4:**
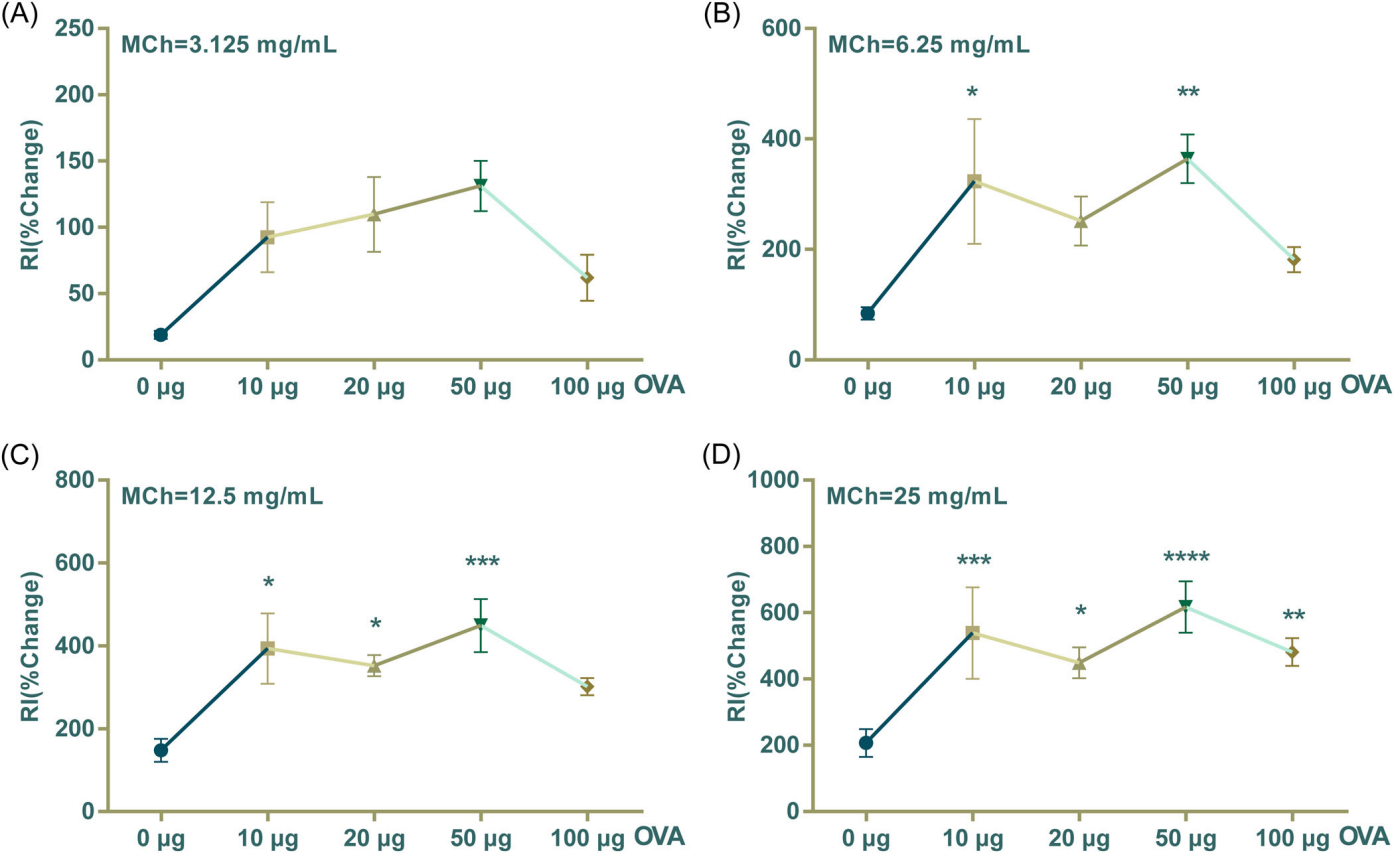
Effect of increasing sensitized ovalbumin (OVA) on airway hyperresponsiveness in mouse asthma model under methacholine (MCh) exposition. (A) The resistance index (RI) of OVA groups when exposed to MCh at the concentration of 3.125 mg/mL. (B) The RI of OVA groups when exposed to MCh at the concentration of 6.25 mg/mL. (C) The RI of OVA groups when exposed to MCh at the concentration of 12.5 mg/mL. (D) The RI of OVA groups when exposed to MCh at the concentration of 25 mg/mL. The values are presented as mean ± SEM (*n* = 6/group expect *n* = 5 in OVA 10 μg group). *p < .05; **p < .01; ***p < .001; ****p < .0001 versus the NS group. *p* < .05 was considered as statistically significant.

However, the impact of sensitive doses of OVA ranging from 10 to 100 μg remains unclear. In this study, we examined the effect of increasing sensitizing OVA doses (0, 10, 20, 50, and 100 μg) on AHR in asthmatic mice. Our findings are consistent with previous research indicating that antigen dosage can influence murine AHR and suggest that sensitization with a dose of 10 μg may be more effective than using a dose of 100 μg. Furthermore, our findings indicate that the dose of 50 μg is significantly superior to both 10 and 100 μg in terms of its effect on OVA‐induced AHR. Additionally, we observed a nonlinear relationship between the sensitive doses of OVA and AHR, which resembles a bell‐shaped curve. With each concentration of MCh in the study, there was an initial increase followed by a decrease in airway resistance with increasing sensitive doses of OVA (Figure 4). Therefore, we propose the concept of effective interval dosage and threshold dose for OVA‐induced asthma sensitization, and suggest that a dosage of approximately 50 μg is most effective in inducing maximum AHR. However, this dosage is suitable for inducing the highest level of AHR. In situations where the airway constriction or relaxation effects of the research objects are ambiguous, it may be necessary to adjust the dosage accordingly.

Exposure to allergens typically occurs at low concentrations. Experimental animal studies have demonstrated that high dose allergen exposure independent of the route of administration favors immune tolerance, while low‐dose allergen exposure favors immune responsiveness.^21^ In vitro studies have revealed that elevated levels of antigen appear to selectively stimulate Th1 cells which produce IFN‐γ and reduce activation of IL‐4‐producing Th2 cells.^22^ Furthermore, studies have reported limitations of the intraperitoneal route of sensitization in inducing tolerance, particularly with protein antigens like OVA.^23^ This may explain why a 100 μg OVA sensitizing dose resulted in lower AHR compared to lower doses.

It has been previously reported that AHR is more elevated in the BALB/c background compared to C57BL/6 mice, while C57BL/6 mice exhibit higher levels of eosinophilia than BALB/c mice in response to OVA.^24^ In this study, a significant elevation of AHR was observed in the BALB/c asthma model sensitized with various doses of OVA, whereas only a significant increase in eosinophils was found in the OVA 50 μg group. The results of this study are consistent with previous findings. Notably, we observed a similar pattern in the changes of airway resistance and total cells count, as well as lymphocytes, eosinophils and basophils upon increasing doses of OVA sensitization: an initial increase followed by a decrease beyond a certain threshold dose. However, this phenomenon was not observed in macrophages. Moreover, the absence of correlation between H&E or PAS score and AHR in this study implies that these may be two mechanistically independent events occurring in lung tissues and BALF during allergic airway diseases, as delineated by the models presented.

In the study, we found an increase in the number of basophils in BALF. There is a plethora of information from murine model regarding the role of basophils in asthma.^25^ Also, studies have reported that basophils are increased in the sputum of patients with eosinophilic asthma compared to those with noneosinophilic asthma.^26^, ^27^ Basophils have been identified as key regulators of allergy in vivo, including orchestrating Th2 immunity to protease allergens in the induction phase.^28^ And it has been reported that basophil‐derived IL‐4 enhances expression of the chemokine CCL11, as well as IL‐5, IL‐9, and IL‐13 in natural helper cells, thus attracting eosinophils.^29^ Furthermore, eosinophilic tissue infiltration, mediated by IL‐4, is attributed to basophils.^26^ However, the physiological purpose of basophils in asthma also remains elusive, as well as their interactions with other effector cells of the allergic inflammatory responses.

Additionally, it is important to acknowledge the limitations of this study. For instance, the animals utilized for collecting BALF were also used to measure AHR with MCh. It should be noted that MCh reactive subjects may exhibit increased cell counts and elevated neutrophil levels, which must be taken into consideration during data analysis. Besides, we observed an increased lymphocyte count in the BALF, which is consistent with a study that employed an Auto Hematology Analyzer for cellular classification.^19^ We postulated that the absence of erythrocytes lysis in BALF samples during the experiment might have led to this outcome,^30^ with the instrument erroneously identifying mouse erythrocytes as lymphocytes.

## 5 CONCLUSIONS

In summary, this study investigated the impact of increasing sensitizing doses of OVA in a murine model of asthma. The present findings are consistent with previous research indicating that antigen dosage can influence airway resistance development and cell count in BALF. Additionally, we propose that there exists an optimal range for OVA sensitization dosage and a threshold dose for inducing asthma symptoms. Based on our results, the optimal OVA dosage for inducing maximal AHR is 50 μg. However, for practical applications, it is recommended to carefully determine the appropriate level of OVA sensitization based on specific experimental requirements, taking into account not only AHR but also other aspects of immune responses. Moreover, our limited data suggests that the alteration of AHR in response to sensitizing OVA doses may be associated with cells present in BALF rather than those found in lung tissues. In future studies, it is necessary to further investigate the effects of different challenging OVA doses on AHR, IgE and cytokine production patterns as well as other parameters involved in mouse asthma modeling protocols.

## AUTHOR CONTRIBUTIONS

Yong‐Qing Yang designed the study and critically reviewed the manuscript. Yan‐Jiao Chen executed, interpreted the data, analysis the data, drafted the manuscript. All the authors have made noteworthy contributions to the study design, data collection, review and interpretation; have engaged in the drafting or revision of the article; have agreed to submit to the current journal; have given final approval of the version to be published; and have agreed to be responsible for all aspects of the work.

## CONFLICT OF INTEREST STATEMENT

The authors declare no conflict of interest.

## ETHICS STATEMENT

The animal procedures were reviewed and approved by the Committee on the Ethics of Animal Experiments of Shanghai University of Traditional Chinese Medicine and handled according to the recommendations of the National Institutes of Health Guide for Care and Use of the Laboratory Animals (Publication No. 8023, revised 1978). Ethical approval number: PZSHUTCM 210320002. The manuscript's final version underwent review by all authors and received their consent for submission.

## Data Availability

The data that support the findings of this study are available from the corresponding author upon reasonable request.
